# Alternative polyadenylation trans-factor FIP1 exacerbates UUO/IRI-induced kidney injury and contributes to AKI-CKD transition via ROS-NLRP3 axis

**DOI:** 10.1038/s41419-021-03751-3

**Published:** 2021-05-19

**Authors:** Tong Zheng, Yuqin Tan, Jiang Qiu, Zhenwei Xie, Xiao Hu, Jinhua Zhang, Ning Na

**Affiliations:** 1 Department of Kidney Transplantation, The Third Affiliated Hospital of Sun Yat-sen University, Guangzhou, Guangdong China; 2 Department of Organ Transplantation, The First Affiliated Hospital of Sun Yat-sen University, Guangzhou, Guangdong China

**Keywords:** Acute kidney injury, Inflammasome

## Abstract

NLRP3, a decisive role in inflammation regulation, is obviously upregulated by oxidative stress in kidney injury. The NLRP3 upregulation leads to unsolved inflammation and other pathological effects, contributing to aggravation of kidney injury and even transition to chronic kidney disease (CKD). However, the mechanism for NLRP3 upregulation and further aggravation of kidney injury remains largely elusive. In this study, we found *NLRP3* 3′UTR was shortened in response to kidney injury in vivo and oxidative stress in vitro. Functionally, such *NLRP3* 3′UTR shortening upregulated NLRP3 expression and amplified inflammation, fibrogenesis, ROS production and apoptosis, depending on stabilizing *NLRP3* mRNA. Mechanistically, FIP1 was found to bind to pPAS of *NLRP3* mRNA via its arginine-rich domain and to induce *NLRP3* 3′UTR shortening. In addition, FIP1 was upregulated in CKD specimens and negatively associated with renal function of CKD patients. More importantly, we found FIP1 was upregulated by oxidative stress and required for oxidative stress-induced NLRP3 upregulation, inflammation activation, cell damage and apoptosis. Finally, we proved that FIP1 silencing attenuated the inflammation activation, fibrogenesis, ROS production and apoptosis induced by UUO or IRI. Taken together, our results demonstrated that oxidative stress-upregulated FIP1 amplified inflammation, fibrogenesis, ROS production and apoptosis via inducing 3′UTR shortening of *NLRP3*, highlighting the importance of crosstalk between oxidative stress and alternative polyadenylation in AKI-CKD transition, as well as the therapeutic potential of FIP1 in kidney injury treatment.

## Introduction

Acute and chronic kidney injury, generally caused by ischemia, obstructive pathologies and chemical drug, have become a major health concern worldwide with high mortality and morbidity^1^. The long-term ending of kidney injury is chronic kidney disease (CKD) and even kidney failure. Unfortunately, there remain no effective drugs for kidney injury^2,3^.

In kidney injury, oxidative stress and inflammation are the most significant characteristics, as well as pivotal pathological factors^4^. In response to injury, inflammation initially emerges as a reno-protective role. However, amplified inflammation, especially induced by oxidative stress, turns into pro-injury effects in multiple ways^5,6^, making inhibition of amplified inflammation as a promising therapeutic strategy of kidney injury^7–9^.

NLRP3, the master component of inflammasome, is the central driver of inflammation^10^. Moreover, aberrant NLRP3 upregulation leads to overactivated inflammation and even CKD^11,12^. Although some biological events, especially the generation of reactive oxygen species (ROS), have been found to account for NLRP3 activation, the exact mechanism for disease-causing NLRP3 overactivation is still obscure^13^. On the other hand, none of NLRP3 inhibitors which have been proved effective in animal experiments is approved clinically, at least partly due to broad immunosuppressive side effects^14^. Therefore, to uncover the mechanism of NLRP3 overactivation is in great need, for improving NLRP3-targeted therapy.

Recently, the length change of mRNA 3′ untranslated regions (3′UTR), derived from alternative polyadenylation (APA), has been identified as an emerging mechanism of oncogene overactivation^15^. Functionally, APA determines mRNA stability and translational efficiency mainly via regulating the response of mRNA to microRNA-mediated degradation, thus making a difference in final protein expression^16,17^. Considering microRNA’s indispensable role in maintaining the homeostasis of NLRP3 expression^18–20^, APA may be an unrecognized contributor to NLRP3 overactivation, possibly offering better strategy for NLRP3-targeted therapy and even kidney injury treatment.

The goal of this study is to better understand the mechanism of inflammation overactivation upon kidney injury and whether APA participates in this process. Here we showed 3′UTR of *NLRP3* mRNA is distinctly shortened in response to ischemia-reperfusion injury (IRI)/ unilateral ureteral obstruction (UUO) in vivo and to oxidative stress in vitro. Such 3′UTR shortening amplifies inflammation, fibrogenesis, ROS production and apoptosis in kidney tubular epithelial cells via enhancing mRNA stability and protein expression of NLRP3. Mechanistically, we found the APA trans-factor FIP1 binds to proximal PAS of *NLRP3* 3′UTR via its arginine-rich domain and induces *NLRP3* 3′UTR shortening. Moreover, obvious FIP1 upregulation is observed in both UUO/IRI kidney injury models and clinical CKD specimens. In particular, we demonstrated that oxidative stress induces such FIP1 upregulation and subsequent 3′UTR shortening of *NLRP3*. Finally, we confirmed FIP1 knockdown alleviates the inflammation, fibrogenesis, ROS production and apoptosis induced by UUO or IRI. Thus, APA regulation is a key mechanism of NLRP3 overactivation and unsolved inflammation in kidney injury. To inhibit 3′UTR shortening-induced NLRP3 overactivation via suppressing FIP1, is a promising strategy for NLRP3-targeted therapy and kidney injury treatment.

## Material and methods

### Cell culture and hypoxia/reoxygenation (H/R) stimulation

Tubular epithelial cell line HK-2 was a kind gift from Dr. Liutao Chen (Sun Yat-sen University). Tubular epithelial cell line HKC was purchase from cell bank of Chinese Academy of Medical Sciences. Cells were grown in DMEM/F-12 medium containing 10% fetal bovine serum (FBS) and incubated at 37 °C in a humidified atmosphere with 5% CO_2_. To induce H/R model, cells were cultured in hypoxia (1% O_2_, 94% N_2_, and 5% CO_2_) for 12 hours. And then, cells were cultured in normoxia (5% CO_2_ and 95% air) for 6 hours. The control cells were incubated in normoxia (5% CO_2_ and 95% air). Cells were routinely tested for Mycoplasma contamination.

### Western blot

Immunoblot assays were performed according to standard protocol. Target proteins were detected with antibodies against NLRP3 (A12694, Abclonal, Wuhan, China), collagen I (A5786, Abclonal, Wuhan, China), fibronectin (A16678, Abclonal, Wuhan, China), Flag (F1804, Sigma, St Louis, USA), caspase1 (A0964, Abclonal, Wuhan, China), IL-1β (A11369, Abclonal, Wuhan, China), IL-18 (A1115, Abclonal, Wuhan, China), phos-smad3 (AP0727, Abclonal, Wuhan, China), E-cadherin (3195, CST, Danvers, USA), N-cadherin (14215, CST, Danvers, USA), FIP1 (A7138, Abclonal, Wuhan, China), β-actin (AC026, Abclonal, Wuhan, China).

### Tissue specimens and immunohistochemical (IHC) staining

The clinical tissue specimens of renal fibrosis were obtained from The First Affiliated Hospital of Sun Yat-sen (Guangzhou, China). IHC staining was performed as previously described^21^. Target proteins were detected with antibodies against NLRP3 (Servicebio, Wuhan, China), FIP1 (Abclonal, Wuhan, China), collagen I (Sevicebio, Wuhan, China), Fibronectin (Servicebio, Wuhan, China), CD3 (Servicebio, Wuhan, China) and F4/80 (Servicebio, Wuhan, China) and evaluated by two independent examiners who were blinded to the animal groups.

### qRT-PCR and the quantification of usage of distal PAS

Total RNA was extracted from cells with Trizol reagent (Invitrogen, Carlsbad, USA) and reversely transcribed into cDNA with SuperScript III Reverse Transcriptase Kit (Invtriogen). The qRT-PCR assays were performed with SYBR-GREEN (Roche, Basel, Switzerland). A method which was previously described^22,23^ was used to quantify the usage of distal PAS. A pair of primers targeting the ORF was designed to represent the total transcripts, and another pair of primers targeting sequences just before the distal PAS (dPAS) to represent the long transcripts. The percentage of dPAS usage was calculated as follows: ΔCt_total_ (ΔCt_distal_) = Ct_total_ (Ct_distal_) − Ct_GAPDH_. ΔΔCt = ΔCt_distal_ − ΔCt_total_. Data are presented as the fold change normalized to the control, calculated as follows: ΔΔΔCt = ΔΔCt_average_ target − ΔΔCt_average of control_. Then the increase or decrease in dPAS usage was calculated as ±2^-normalized ΔΔΔCt^. The negative value indicated that the mRNA had 3′UTR shortening compared with the control. All primer sequences are listed in Supplementary Table 1.

### Rapid amplification of 3′cDNA ends (3′RACE) assay

The NLRP3 cDNA was generated with 3′Full Race Core Set (Takara, Kyoto, Japan). The first round PCR was performed with NLRP3 GSP-1 primer and 3′RACE outer primer. The nested PCR was performed with NLRP3 GSP-2 primer and 3′RACE inner primer. The products of PCR were cloned with TA/Blunt-Zero cloning kit (Vazyme, Wuhan, China) and further sequenced. All primer sequences are listed in Supplementary Table 1.

### Luciferase reporter assay

The short-3′UTR and long-3′UTR of *NLRP3* were cloned downstream of the Renilla luciferase in psiCHECK-2. After transfection for 48 hours, luciferase activity was tested using the Dual-Luciferase Reporter Assay Kit (Promega, Madison, USA).

### Unilateral ureteral obstruction (UUO) and ischemia-reperfusion injury (IRI) kidney injury model

8-week-old C57/BL6 male mice were used and randomly divided into groups as needed for kidney injury animal model. For UUO model, mice were anesthetized with pentobarbital sodium (50 mg/kg) and placed on a homeothermic table to maintain body temperature at 37 °C. Midline incision was made to open the abdomen of mice. Left ureter was ligated at proximal and distal points, and the ureter between ligated points was cut off. The abdomen was closed in layers. 14 days later, mice were sacrificed and the kidneys were collected. For IRI model, briefly, after a right nephrectomy, the left kidney was subjected to ischemia for 30 minutes with a non-traumatic vascular clamp, follow by 48-hour reperfusion. Sham control animals were subjected to the identical operation without ischemia.

The serum or plasma was collected and stored at −80 °C for further analysis. The kidney tissues were fixed in 4% paraformaldehyde (PFA) for histology analysis. The remaining kidney tissue was stored at −80 °C for biochemical analysis.

### Measurement of ROS production

The ROS generation in cells and kidney tissues was detected with fluorescent probes DHE (KEYGEN Bio, Nanjing, China) and DCFH-DA (KEYGEN Bio, Nanjing, China). The cells or frozen specimens were incubated with DHE or DCFH-DA for 30 minutes. The fluorescent signal was captured with NIS elements and quantified with Image-pro Plus 6.0.

### Measurement of oxidative stress

The commercial kits were purchased from Nanjing Jiancheng Bio (Nanjing, China) to measure oxidative stress in vivo and in vitro. The activity of superoxide dismutase (SOD, A001–3, hydroxylamine method) and concentration of malondialdehyde (MDA, A003–1, thiobarbituric acid method) were measured according to manufacturer’s instruction.

### TUNEL assay

The apoptosis in cells and kidney tissues was examined with commercial kit (Vazyme, Wuhan, China). Briefly, the samples were fixed and then exposed to the TUNEL reaction mixture containing TM red–labeled dUTP. After that, samples were counterstained with DAPI. TUNEL-positive nuclei were identified with fluorescence microscopy.

### RNA immunoprecipitation (RIP)

10^8^ HK2 cells were used to extract total proteins with lysis buffer (40 mM Tris, 120 mM NaCl, 1% Triton X-100, 1 mM NaF, 1 mM Na_3_VO_4_, 1 × protease inhibitor cocktail and 1 U/ml RNasin inhibitor). Protein A/G beads were incubated with Flag antibody and then used to isolate FIP1 protein, along with the FIP1-binding RNAs. TRIzol reagent was used to extract the co-precipitated RNAs. The amount of *NLRP3* mRNA was analyzed by qRT-PCR.

### AAV9-mediated *Fip1* knockdown in mice

The plasmids pAAV2/9, pHelper and pAAV-ZsGreen1-shRNA were obtained from Youbio company (Changsha, China). A *Fip1* shRNA oligo was cloned into a pAAV-ZsGreen1-shRNA vector. These plasmids were transfected into HEK293 to develop the AAV9 expressing *Fip1* shRNA or control shRNA. Then mice were anesthetized and 100 μl AAV9 (1 × 10^11^ viral genome particles) was injected slowly into the renal vein using a 31 G needle^24^. The shRNA sequences were listed as follows:*Fip1* shRNA sense (5′-3′): GATCCCACTGAAGTAGACAACAATTCTCGAGAATTGTTGTCTACTTCAGTGGTTTTTG

Control shRNA sense (5′-3′): GATCCTTCTCCGAACGTGTCACGTCTCGAGACGTGACACGTTCGGAGAATTTTTG

### Statistical analysis

In each experiment, data were presented as the mean ± S.E.M. Student’s t test was used to analysis the statistical significance between groups. The person correlation test was used to analysis the correlation. Data were performed with GraphPad Prism 8.0.

## Results

### 3′UTR of *NLRP3* is shortened in response to kidney injury

**Fig. 1:**
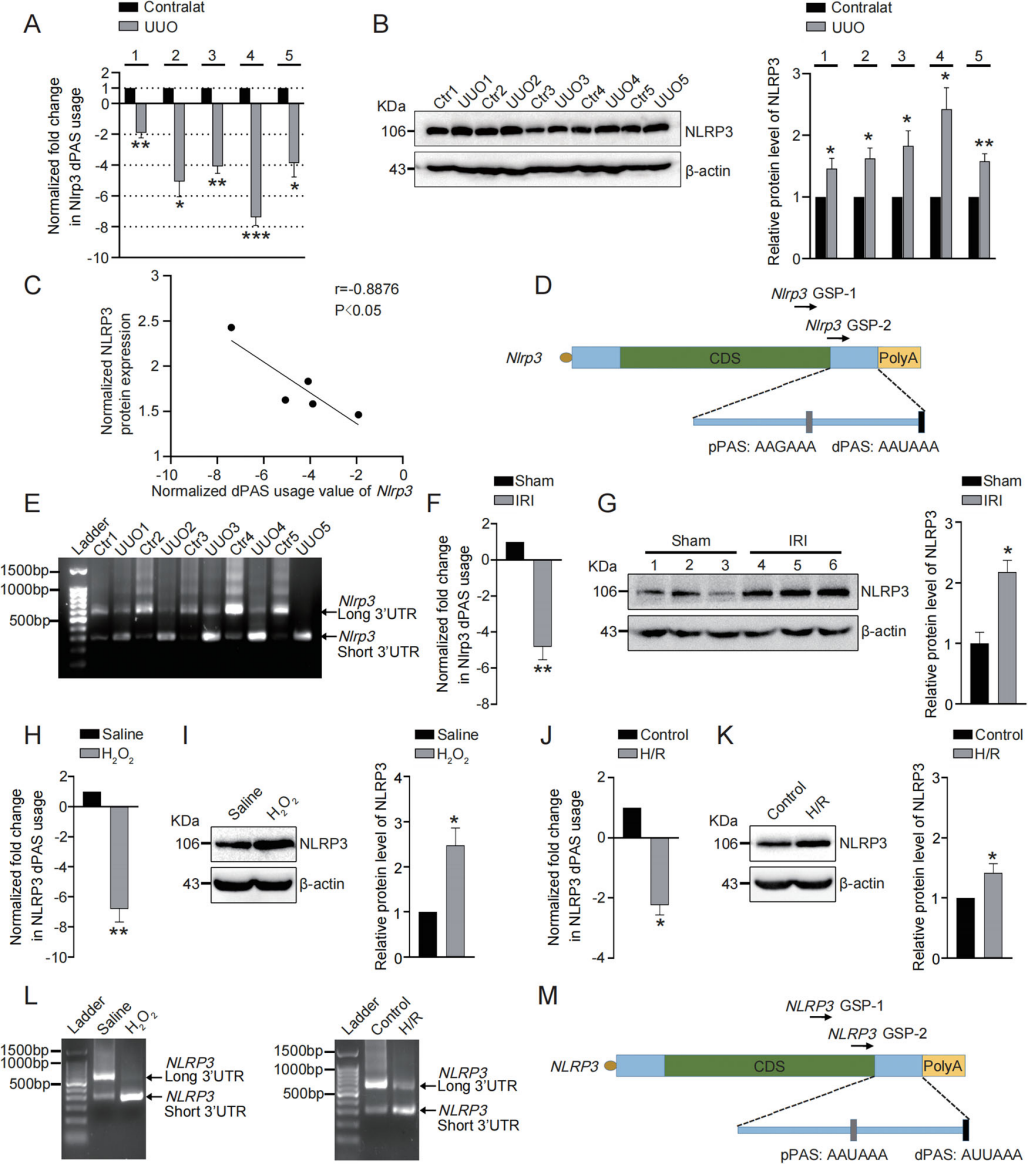
3′UTR of *NLRP3* is shortened in response to kidney injury. **A** qRT-PCR was performed to determine the normalized fold changes of distal PAS usage of *NLRP3* in UUO kidneys (*n* = 5) compared to normal kidneys (*n* = 5). **B** Western blot was performed and quantitatively analyzed to determine the protein levels of NLRP3 in UUO kidneys (*n* = 5) compared to normal kidneys (*n* = 5). **C** Correlation of normalized dPAS usage value of *NLRP3* with normalized protein levels of NLRP3. **D** The schematic graph indicating the novel pPAS in mice and the primer sequences used in 3′RACE assay. Green box, CDS; blue bar, UTR; gray box, the newly-identified pPAS; black box, canonical dPAS. **E** 3′RACE was performed to determine the expression of mRNA isoforms of *NLRP3* in UUO kidneys (*n* = 5) compared to normal kidneys (*n* = 5). **F** qRT-PCR was performed to determine the normalized fold changes of distal PAS usage of *NLRP3* in IRI kidneys (*n* = 3) compared to normal kidneys (*n* = 3). **G** Western blot was performed and quantitatively analyzed to determine the protein levels of NLRP3 in IRI kidneys (*n* = 3) compared to normal kidneys (*n* = 3). **H** qRT-PCR was performed to determine the normalized fold changes of distal PAS usage of *NLRP3* in HK2 cells treated with H_2_O_2_. **I** Western blot was performed and quantitatively analyzed to determine the protein levels of NLRP3 in HK2 cells treated with H_2_O_2_. **J** qRT-PCR was performed to determine the normalized fold changes of distal PAS usage of *NLRP3* in HK2 cells treated with H/R. **K** Western blot was performed and quantitatively analyzed to determine the protein levels of NLRP3 in HK2 cells treated with H/R. **L** 3′RACE was performed to determine the expression of mRNA isoforms of *NLRP3* in HK2 cells treated with H_2_O_2_ or H/R. **M** The schematic graph indicating the novel pPAS in human and the primer sequences used in 3′RACE assay. Green box, CDS; blue bar, UTR; gray box, the newly-identified pPAS; black box, canonical dPAS. *P < 0.05; **P < 0.01; ***P < 0.001; All data represent the mean ± SEM obtained from three independent experiments. Student’s *t* test.

To investigate whether APA participates in NLRP3 overactivation upon kidney injury, UUO and IRI kidney injury models were used. The degree of difference of 3′UTR usage of *NLRP3* in five pairs of UUO-induced injured kidneys and matched unobstructed kidneys was examined by qRT-PCR. Compared with contralateral kidneys, short 3′UTR isoform of *NLRP3* was obviously upregulated in all obstructed kidneys to different extents, suggesting *NLRP3* 3′UTR shortening as a pathological event in kidney injury (Fig. 1A). Consistent with previous studies, protein levels of NLRP3 were upregulated following UUO (Fig. 1B). Particularly, the degrees of *NLRP3* 3′UTR shortening were positively associated with the degrees of NLRP3 upregulation (Fig. 1C), suggesting 3′UTR shortening as important contributor to NLRP3 upregulation in obstructed kidneys. Next, 3′ rapid amplification of cDNA ends (3′RACE) was performed with primers described in Fig. 1D to determine whether *NLRP3* 3′UTR shortening following UUO derived from usage of novel proximal PAS (pPAS). In all obstructed kidneys, a short 3′UTR isoform of *NLRP3* was stably found (Fig. 1E). Therefore, a novel proximal PAS (pPAS) corresponding to the short 3′UTR isoform of *NLRP3* was identified in mice (Fig. 1D). Similarly, in IRI kidneys, obvious 3′UTR shortening of *NLRP3* was also observed, as well as enhanced protein levels of NLRP3 (Fig. 1F, G).

In order to confirm whether 3′UTR shortening of *NLRP3* existed in human, the human renal proximal tubular epithelial cells HK2 were treated with H_2_O_2_ or hypoxia/reoxygenation (H/R) to simulate UUO (ref. ^25^) or IRI (ref. ^1^) model in vitro. Both *NLRP3* 3′UTR shortening and NLRP3 upregulation were induced by stimulation of H_2_O_2_ or H/R (Fig. 1H–K). Furthermore, 3′RACE was performed and showed that in HK2 cells treated with H_2_O_2_ or H/R, an unreported short 3′UTR isoform of *NLRP3* appeared (Fig. 1L), identifying a novel pPAS in human (Fig. 1M). These results demonstrated *NLRP3* 3′UTR is shortened in response to kidney injury, and such 3′UTR shortening associates with NLRP3 upregulation, suggesting a key role of APA regulation in NLRP3 overactivation and kidney injury progression.

### 3′UTR shortening of *NLRP3* amplifies inflammation, fibrogenesis, ROS production and apoptosis in renal tubular epithelial cells

**Fig. 2:**
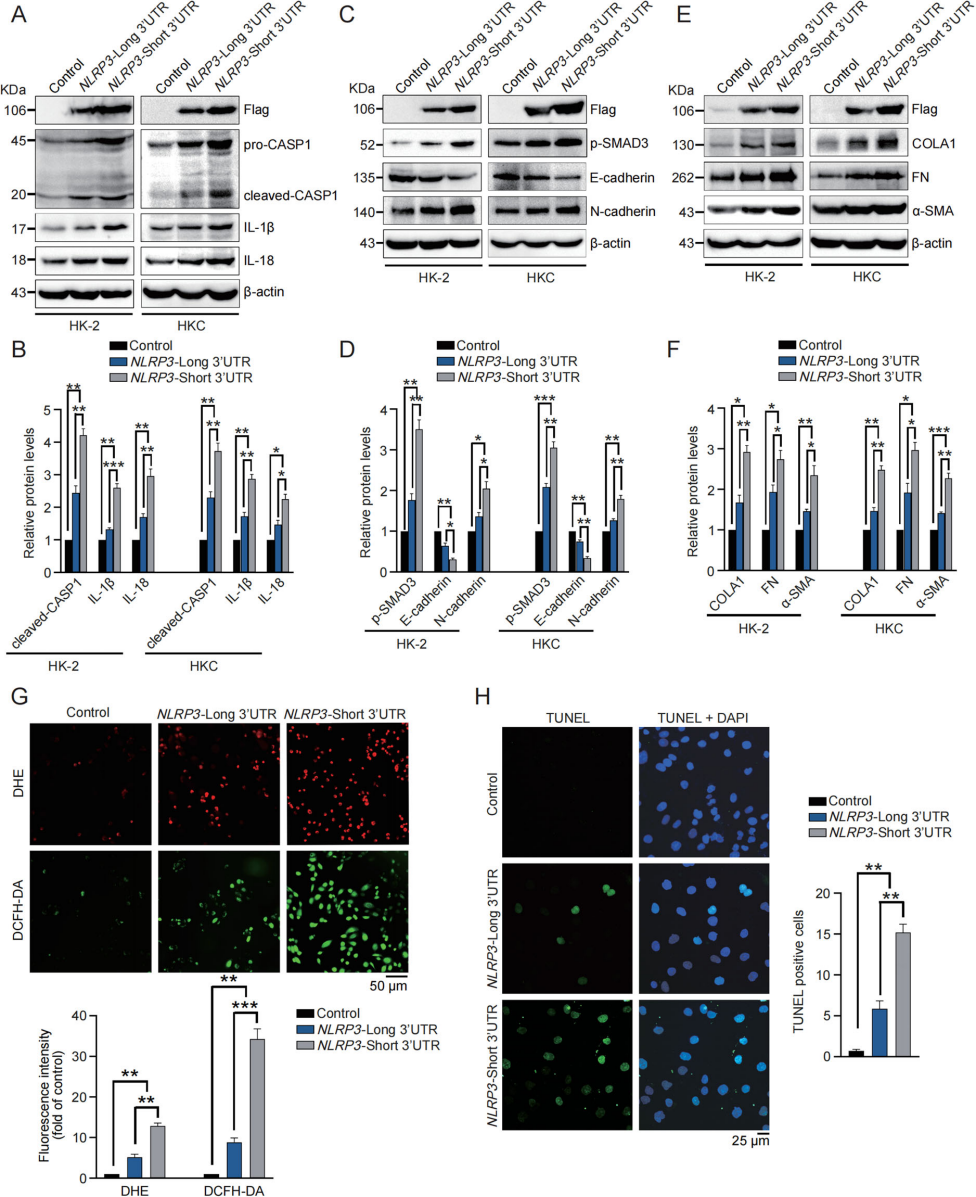
3′UTR shortening of *NLRP3* amplifies inflammation, fibrogenesis, ROS production, and apoptosis in renal tubular epithelial cells. **A** Western blot was performed to determine the protein levels of Flag, caspase1, IL-1β, and IL-18 in HK2 and HKC cells transfected with empty vector, short 3′UTR *NLRP3* or long 3′UTR *NLRP3* respectively. **B** Quantitative analysis of protein levels of caspase1, IL-1β and IL-18 in HK2 and HKC cells transfected with empty vector, short 3′UTR *NLRP3* or long 3′UTR *NLRP3*. **C** Western blot was performed to determine the protein levels of Flag, phospho-SMAD3, E-cadherin, and N-cadherin in HK2 and HKC cells transfected with empty vector, short 3′UTR *NLRP3* or long 3′UTR *NLRP3* respectively. **D** Quantitative analysis of protein levels of phospho-SMAD3, E-cadherin and N-cadherin in HK2 and HKC cells transfected with empty vector, short 3′UTR *NLRP3* or long 3′UTR *NLRP3*. **E** Western blot was performed to determine the protein levels of Flag, collagen I, fibronectin, and α-SMA in HK2 and HKC cells transfected with empty vector, short 3′UTR *NLRP3* or long 3′UTR *NLRP3* respectively. **F** Quantitative analysis of protein levels of collagen I, fibronectin, and α-SMA in HK2 and HKC cells transfected with empty vector, short 3′UTR *NLRP3* or long 3′UTR *NLRP3*. **G** Representative images and quantitative analysis of DHE and DCFH-DA staining in HK2 cells transfected with empty vector, short 3′UTR *NLRP3* or long 3′UTR *NLRP3*. **H** Representative images and quantitative analysis of TUNEL staining in HK2 cells transfected with empty vector, short 3′UTR *NLRP3* or long 3′UTR *NLRP3*. *P < 0.05; **P < 0.01; *** *P* < 0.001; All data represent the mean ± SEM obtained from three independent experiments. Student’s *t* test.

We thus continued to investigate the function of *NLRP3* 3′UTR shortening in vitro. Considering the canonical function of NLRP3 (ref. ^26^), the effects of *NLRP3* 3′UTR shortening in inflammasome activation was examined. HK2 and HKC cells were transfected with newly-identified short-3′UTR isoform of *NLRP3* or long-3′UTR isoform of *NLRP3* (namely full-length *NLRP3*) respectively, and significantly enhanced expression of caspase1, IL-1β and IL-18 was observed in both cells transfected with short-3′UTR *NLRP3*, compared with those transfected with full-length NLRP3 or empty vector (Fig. 2A, B), indicating the amplified inflammasome activation induced by *NLRP3* 3′UTR shortening.

Independently of inflammasome, NLRP3 also functions in fibrogenesis to aggravate kidney injury via activating TGF-β pathway^27^. As evidenced by the stronger stimulation of phosphorylated SMAD3 (Fig. 2C, D), TGF-β pathway was found more activated upon transfection of short-3′UTR *NLRP3*. Accordingly, short-3′UTR *NLRP3* displayed advantages in promoting fibrogenesis (Fig. 2E, F). More importantly, NLRP3 was reported to trigger ROS production, and DHE/DCFH-DA staining showed ROS production benefited from *NLRP3* 3′UTR shortening (Fig. 2G). Finally, we evaluated the effect of *NLRP3* 3′UTR shortening on cell apoptosis and found short-3′UTR *NLRP3* caused more cell apoptosis than the full-length one did (Fig. 2H). These in vitro evidences strongly suggested the pathological role of *NLRP3* 3′UTR shortening in kidney injury.

### 3′UTR shortening of *NLRP3* benefits mRNA stability and leads to enhanced protein expression of NLRP3

**Fig. 3:**
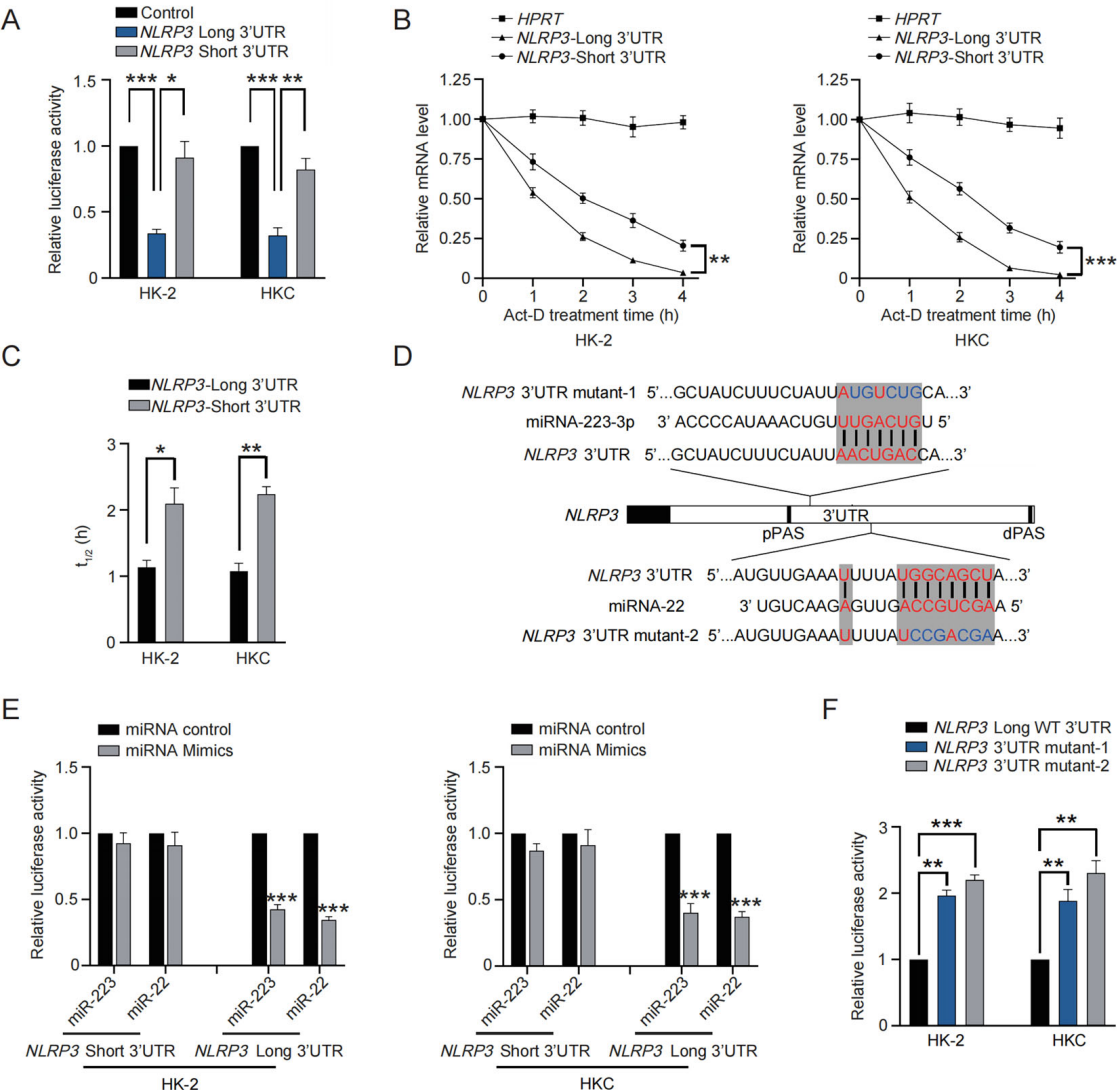
3′UTR shortening of *NLRP3* benefits mRNA stability and leads to enhanced protein expression of NLRP3. **A** Luciferase reporter assay was performed in HK2 and HKC cells to examine luciferase activity produced by reporters carrying short 3′UTR or long 3′UTR of *NLRP3* respectively. **B** Isoform-specific qRT-PCR was performed to determine long-3′UTR and short-3′UTR *NLRP3* mRNA half-life in HK2 and HKC cells treated with Act-D. Total RNA was normalized with *HPRT*. **C** The bar graph showing the half-life in hours calculated for long-3′UTR *NLRP3* and short-3′UTR *NLRP3*. **D** Schematic diagram showing the binding sites of miRNAs in *NLRP3* 3′UTR and the mutant *NLRP3* used in **F**. **E** Luciferase reporter assay was performed to examine luciferase activity produced by reporters carrying short 3′UTR or long 3′UTR of *NLRP3* respectively in HK2 and HKC cells co-transfected with mimics of miR-223 or miR-22. **F** Luciferase reporter assay was performed to examine luciferase activity produced by reporters carrying long 3′UTR of *NLRP3* or mutants of long 3′UTR of *NLRP3* respectively. *P < 0.05; **P < 0.01; ***P < 0.001; All data represent the mean ± SEM obtained from three independent experiments. Student’s *t* test.

Since the pathological role of *NLRP3* 3′UTR shortening probably relied on increasing NLRP3 protein level, we thus started to uncover how 3′UTR shortening dictated the final expression of NLRP3. Luciferase activity assay demonstrated the reporter plasmid with short 3′UTR of *NLRP3* gained an augmented luciferase activity in both cells (Fig. 3A). Furthermore, the mRNA decay time course was performed with a 3′UTR isoform-specific qRT-PCR strategy^28^. A distinct diversity of half-life between short and long 3′UTR of *NLRP3* was observed (Fig. 3B, C), indicating the superiority of short-3′UTR *NLRP3* in mRNA stability.

MicroRNA-mediated silencing plays a decisive role in mRNA stability, so we hypothesized 3′UTR shortening changed the response of *NLRP3* mRNA to microRNAs. miR-223–3p and miR-22 have been frequently reported to target *NLRP3* in various settings^18,19^, and in particular, both of their binding sites on *NLRP3* 3′UTR were downstream of the newly-identified pPAS (Fig. 3D). Luciferase reporter plasmids with short 3′UTR or long 3′UTR of *NLRP3* were co-transfected with mimics of miR-223–3p or miR-22. In both cells, the miR-223–3/miR-22 mimics weakened luciferase activity of long *NLRP3* 3′UTR, in contrast, they showed no significant effects on luciferase activity of short *NLRP3* 3′UTR (Fig. 3E). On the other hand, the luciferase plasmid carrying mutated long 3′UTR of *NLRP3* (Fig. 3D) displayed an enhanced luciferase activity, compared with the plasmid carrying the wild-type (Fig. 3F). Therefore, these data indicated depending on 3′UTR shortening, *NLRP3* mRNA is stabilized via evading miRNA-mediated silencing, leading to NLRP3 upregulation.

### FIP1 binds to *NLRP3* 3′UTR via C-terminal arginine-rich domain and induces 3′UTR shortening of *NLRP3*

**Fig. 4:**
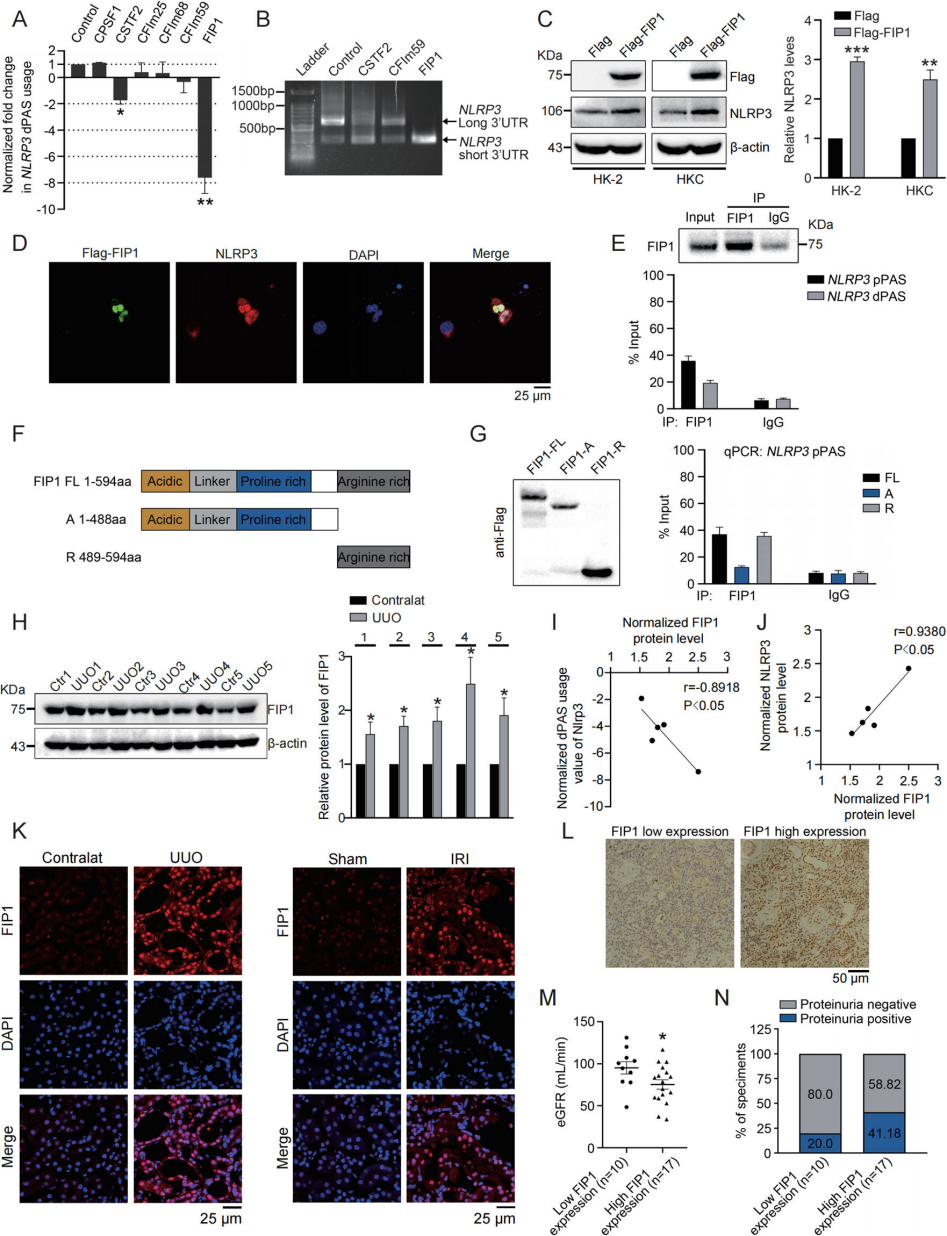
FIP1 binds to *NLRP3* mRNA via C-terminal arginine-rich domain to induce 3′UTR shortening of *NLRP3* and is upregulated in injured kidneys and CKD specimens. **A** qRT-PCR was performed to determine the normalized fold changes of distal PAS usage of *NLRP3* in HK2 cells transfected with each indicated APA trans-factor. **B** 3′RACE was performed to determine the expression of mRNA isoforms of *NLRP3* in HK2 cells transfected with CSTF2, CFIm59, or FIP1 respectively. **C** Western blots were performed and quantitatively analyzed to detect protein levels of NLRP3 in HK2 and HKC cells transfected with Flag-FIP1 or empty vector. **D** Immunofluorescence staining was performed to detect protein levels of NLRP3 in HK2 cells transfected with Flag-FIP1. **E** RNA immonuprecipitation (RIP) assay was performed to determine the region of NLRP3 3'UTR which FIP1binds to. And qRT-PCR was performed to quantify the relative enrichment of *NLRP3* 3′UTR regions associated with FIP1 relative to the input. IgG was used as a control. **F** Schematic diagram showing the truncated regions of FIP1 used for RIP assay. **G** RIP assay was performed to map the binding region of FIP1 to *NLRP3* 3′UTR. And qRT-PCR was performed to quantify the relative enrichment of *NLRP3* mRNA levels associated with each truncated FIP1 relative to the input. IgG was used as a control. **H** Western blot was performed and quantitatively analyzed to determine the protein levels of FIP1 in UUO kidneys (*n* = 5) compared to normal kidneys (*n* = 5). **I** Correlation of normalized protein levels of FIP1 with normalized dPAS usage value of NLRP3. **J** Correlation of normalized protein levels of FIP1 with normalized protein levels of NLRP3. **K** Immunofluorescence staining was performed to detect protein levels of FIP1 in UUO or IRI kidneys compared to normal kidneys. **L** Representative images of IHC staining for FIP1 in FIP1 high-expressed and FIP1 low-expressed CKD specimens. **M** The scatter plot demonstrating the eGFR of patients with high FIP1 expression (*n* = 17) and those with low FIP1 expression (*n* = 10). **N** The correlation analysis between FIP1 expression and proteinuria in CKD patients. **P < 0.01; ***P < 0.001; All data represent the mean ± SEM obtained from three independent experiments. Student’s *t* test.

The events of 3′UTR alteration in most cases are the results of aberrant expression of APA trans-factors^23^, including CSTF2, CPSF1, FIP1, CFIm59, CFIm25 and PABPN1. Therefore, in order to determine the factor governing *NLRP3* 3′UTR shortening, the correlations between the 3′UTR length of *NLRP3* and transfection of the above-mentioned APA trans-factors were examined. In HK2 cells FIP1 was found to predominantly induce *NLRP3* 3′UTR shortening (Fig. 4A). Moreover, only CSTF2 (ref. ^23^), FIP1 (ref. ^29^) and CFIm59 (ref. ^30^) have been reported to induce 3′UTR shortening, then 3′RACE was performed and showed among three candidates, FIP1 gained the strongest capability in upregulating short 3′UTR isoform of *NLRP3* (Fig. 4B), confirming FIP1 as upstream governor of *NLRP3* 3′UTR shortening. Furthermore, western blot and immunofluorescence (IF) staining showed FIP1 upregulated NLRP3 expression in HK2 cells (Fig. 4C, D).

In order to confirm FIP1 as the bona fide inducer of *NLRP3* 3′UTR shortening, RNA immunoprecipitation (RIP) was used to determine which region of *NLRP3* 3′UTR FIP1 binds to. As demonstrated by qRT-PCR, FIP1 was found to predominantly associate with the region around *NLRP3* pPAS, rather than that around dPAS (Fig. 4E). We continued to map the functional domain of FIP1 mediating the binding between FIP1 and *NLRP3* mRNA. The C-terminal arginine-rich domain (Fig. 4F) was previously reported to mediate the binding of FIP1 and mRNA^31^, therefore two recombined truncated Flag-FIP1 (1–488 aa and 489–594 aa) were purified and used for RIP. The RIP assay confirmed FIP1 depended on its arginine-rich (R) domain to bind with *NLRP3* mRNA (Fig. 4G). The above results clearly demonstrated FIP1 as the inducer of NLRP3 3′UTR shortening.

### FIP1 is upregulated in injured kidneys and CKD specimens

We thus studied the role of FIP in kidney injury. To start with, the expression of FIP1 in the five pairs of UUO kidneys and matched unobstructed kidneys which were previously used, was examined. As shown by western blot, FIP1 was overexpressed in UUO kidneys (Fig. 4H). Moreover, the expression of FIP1 was positively associated with *NLRP3* 3′UTR shortening and NLRP3 expression (Fig. 4I, J). Additionally, IF imaging again confirmed the overexpression of FIP1 in both UUO-induced and IRI-induced injured kidneys (Fig. 4K).

Since aggravated kidney injury is very likely to turn into CKD, we investigated the expression of FIP1 in clinical CKD specimens to determine whether FIP1 is a contributor of AKI-CKD transition. The expression of FIP1 in 27 kidney specimens from CKD patients was examined by immunohistochemistry (IHC) staining (Fig. 4L), and these specimens were divided into a group of high FIP1 expression (*n* = 17) and another group of low FIP1 expression (*n* = 10). Importantly, high FIP1 expression negatively associated with eGFR (*P* < 0.05; Fig. 4M). Besides, the patients with high FIP1 expression seemed to have a relatively higher percentage of proteinuria than those with low FIP1 expression did (Fig. 4N). Taken together, these results suggested FIP1 as a harmful role in kidney injury and contributor of AKI-CKD transition.

### FIP1 promotes inflammation, fibrogenesis, ROS production and apoptosis in renal tubular epithelial cells

**Fig. 5:**
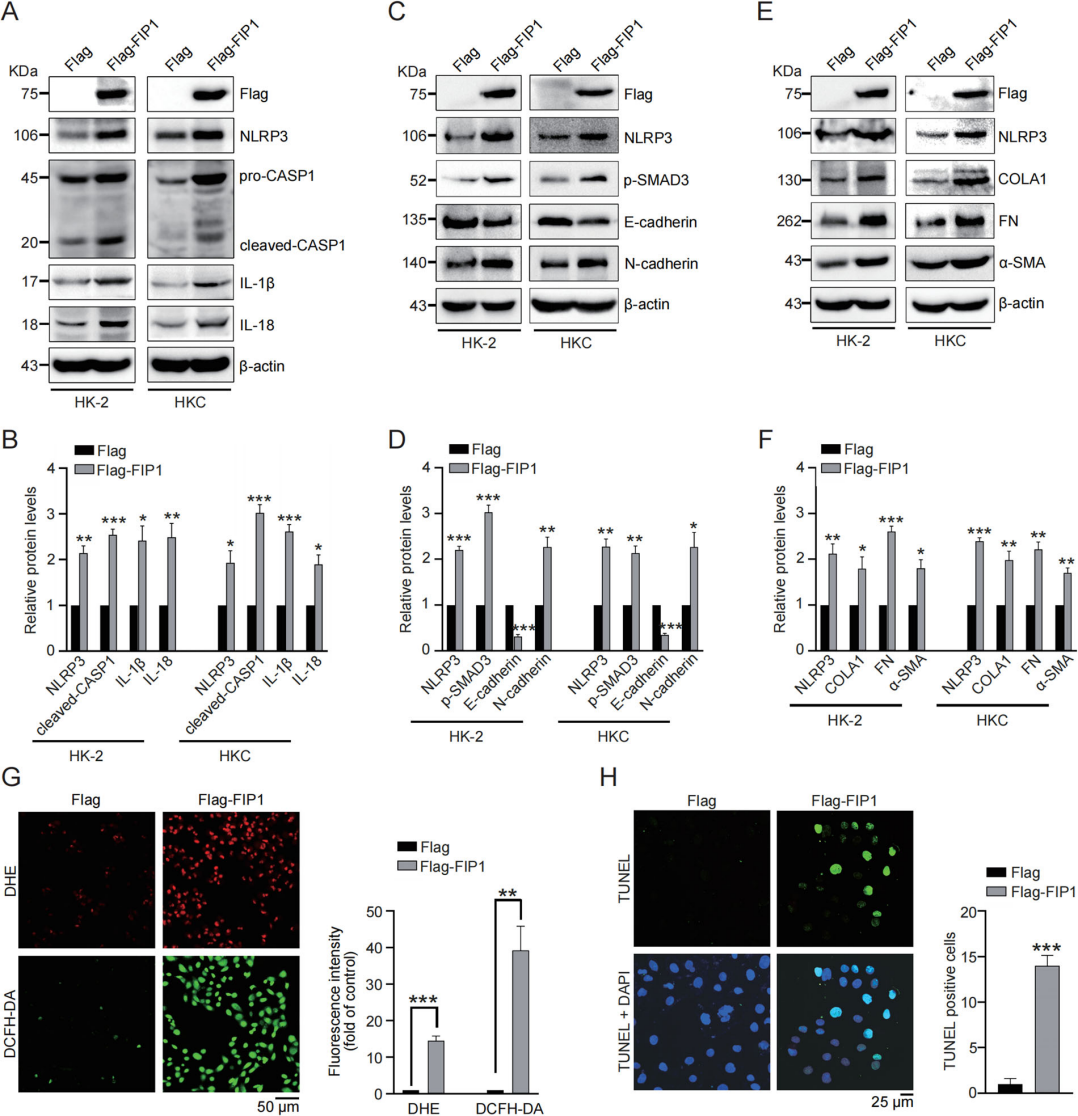
FIP1 promotes inflammation, fibrogenesis, ROS production, and apoptosis in renal tubular epithelial cells. **A** Western blot was performed to determine the protein levels of Flag, caspase1, IL-1β, and IL-18 in HK2 and HKC cells transfected with empty vector or Flag-FIP1 respectively. **B** Quantitative analysis of protein levels of caspase1, IL-1β and IL-18 in HK2 and HKC cells transfected with empty vector or Flag-FIP1 respectively. **C** Western blot was performed to determine the protein levels of phospho-SMAD3, E-cadherin, and N-cadherin in HK2 and HKC cells transfected with empty vector or Flag-FIP1 respectively. **D** Quantitative analysis of protein levels of phospho-SMAD3, E-cadherin, and N-cadherin in HK2 and HKC cells transfected with empty vector or Flag-FIP1 respectively. **E** Western blot was performed to determine the protein levels of collagen I, fibronectin, and α-SMA in HK2 and HKC cells transfected with empty vector or Flag-FIP1 respectively. **F** Quantitative analysis of protein levels of collagen I, fibronectin, and α-SMA in HK2 and HKC cells transfected with empty vector or Flag-FIP1 respectively. **G** Representative images and quantitative analysis of DHE and DCFH-DA staining in HK2 cells transfected with empty vector or Flag-FIP1 respectively. **H** Representative images and quantitative analysis of TUNEL staining in HK2 cells transfected with empty vector or Flag-FIP1 respectively. *P < 0.05; **P < 0.01; ***P < 0.001; All data represent the mean ± SEM obtained from three independent experiments. Student’s *t* test.

Next, the function of FIP1 in renal tubular epithelial cells was investigated. First, transfection of Flag-FIP1 strongly upregulated the expression of caspase1, IL-1β and IL-18, indicating FIP1’s capability of activating inflammasome (Fig. 5A, B). Furthermore, TGF-β pathway and fibrogenesis were stimulated by enforced expression of FIP1 in both cells (Fig. 5C–F). Besides, ROS production was enhanced in HK2 cells by Flag-FIP1 transfection, as shown by DHE and DCFH-DA staining (Fig. 5G). Finally, TUNEL staining confirmed the pro-apoptotic role of FIP1 in HK2 cells (Fig. 5H). Therefore, these results showed that FIP1 behaves as a pro-injury role in kidney tubular cells, suggesting FIP1 as a promising therapeutic target for kidney injury.

### FIP1 is upregulated by oxidative stress and required for oxidative stress-induced NLRP3 upregulation, inflammation, apoptosis and cell damage

**Fig. 6:**
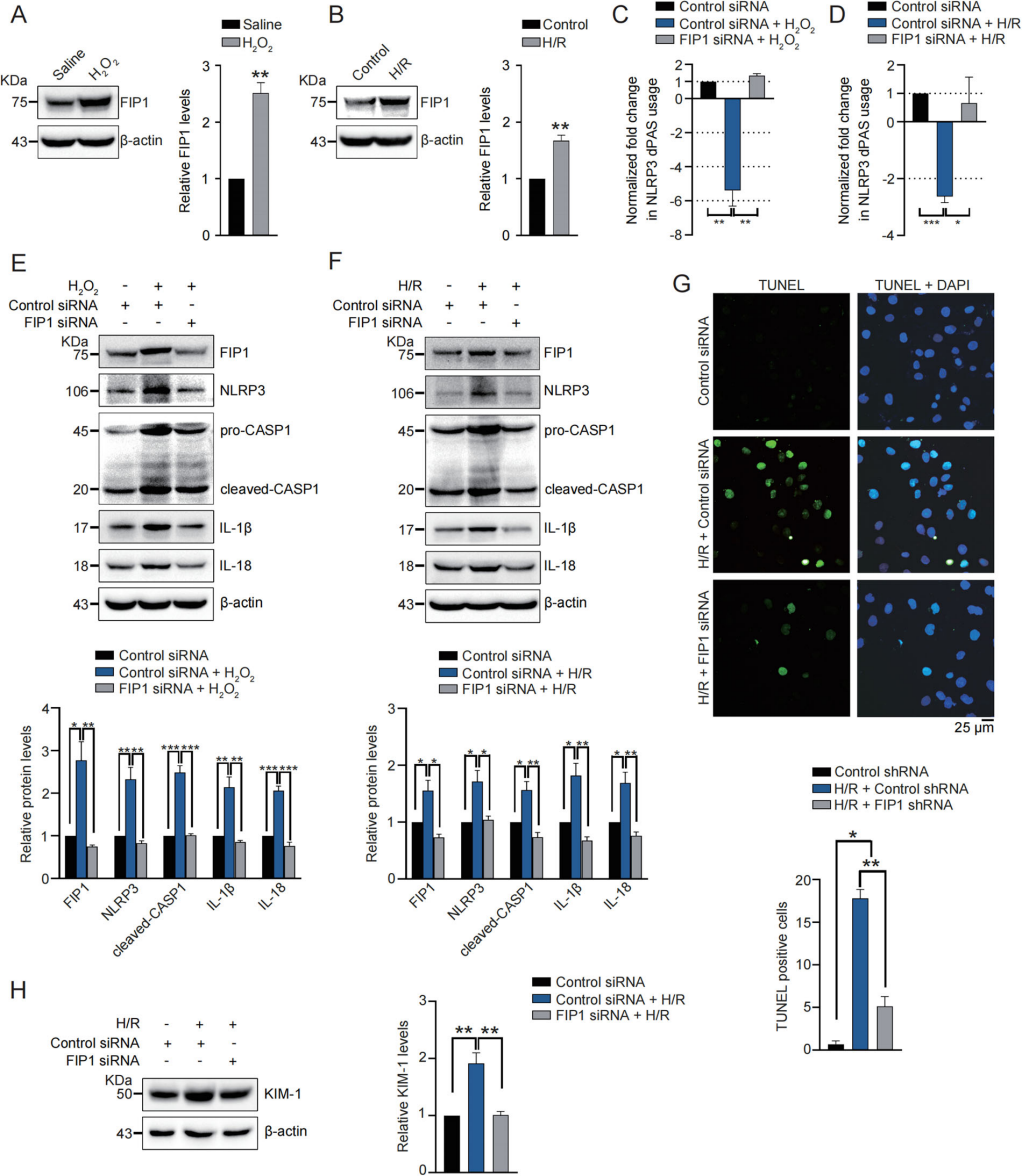
FIP1 is upregulated by oxidative stress and required for oxidative stress-induced NLRP3 upregulation, inflammation, apoptosis, and cell damage. **A** Western blot was performed and quantitatively analyzed to determine the protein levels of FIP1 in HK2 cells treated with saline or H_2_O_2_ (0.5 mM). **B** Western blot was performed and quantitatively analyzed to determine the protein levels of FIP1 in HK2 cells treated with H/R. **C**, **D** qRT-PCR was performed to determine the normalized fold changes of distal PAS usage of *NLRP3* in HK2 cells with indicated treatment. **E**, **F** Western blot was performed and quantitatively analyzed to determine the protein levels of FIP1, NLRP3, caspase1, IL-1β, and IL-18 in HK2 cells with indicated treatment. **G** Representative images and quantitative analysis of TUNEL staining in HK2 cells with indicated treatment. **H** Western blot was performed and quantitatively analyzed to determine the protein levels of KIM-1 in HK2 cells with indicated treatment. *P < 0.05; **P < 0.01; ***P < 0.001; All data represent the mean ± SEM obtained from three independent experiments. Student’s *t* test.

Since we found 3′UTR shortening of *NLRP3* was induced by oxidative stress and governed by APA trans-factor FIP1, we wondered whether oxidative stress functioned upstream of FIP1. We thus treated HK2 cells with H_2_O_2_ or H/R and found FIP1 was obviously upregulated by both treatments (Fig. 6A, B), suggesting oxidative stress may depend on upregulating FIP1 to induce *NLRP3* 3′UTR shortening. To prove this, FIP1 was silenced in HK2 cells, followed by treatment of H_2_O_2_ or H/R. Indeed, FIP1 silencing reversed the 3′UTR shortening of *NLRP3* which was induced by treatment of H_2_O_2_ or H/R (Fig. 6C, D).

In kidney injury, oxidative stress mainly functions through promoting inflammation, apoptosis^6,32^. In particular, oxidative stress acts as a key stimulator of NLRP3 inflammasome activation^33^. We thus hypothesized FIP1 might mediate the oxidative stress-induced NLRP3 inflammasome activation. Significantly, FIP1 silencing abolished NLRP3 upregulation and subsequent inflammasome activation induced by H_2_O_2_ or H/R (Fig. 6E, F). In addition, H/R stimulation-induced tubular cell apoptosis and damage were both mitigated by FIP1 silencing (Fig. 6G, H). These results emphasized the importance of FIP1 and APA regulation in oxidative stress-induced kidney injury.

### Knockdown (KD) of FIP1 alleviates UUO-induced fibrogenesis, inflammation activation, oxidative stress and apoptosis

**Fig. 7:**
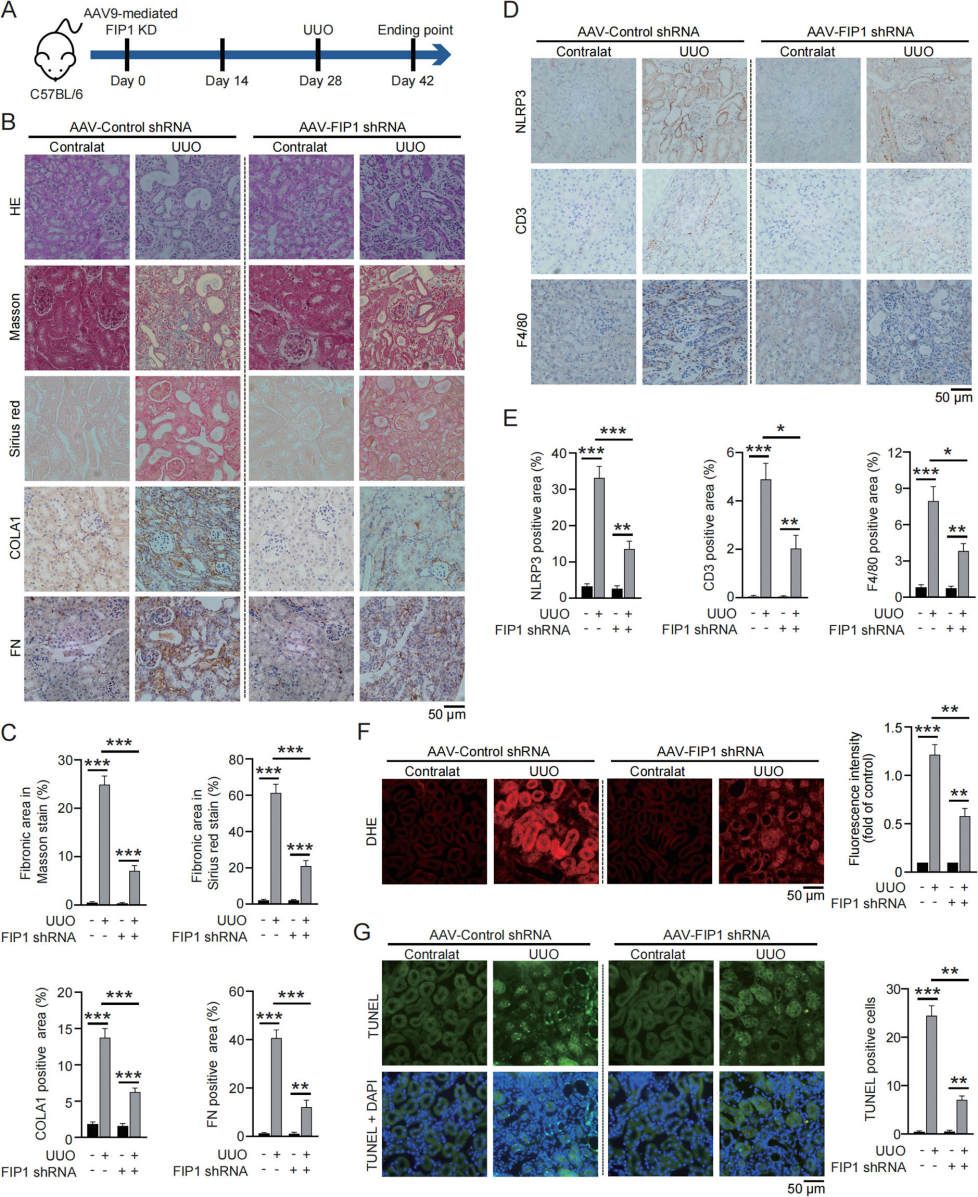
FIP1 KD alleviates UUO-induced fibrogenesis, inflammation activation, oxidative stress, and apoptosis. **A** Schematic diagram demonstrating the animal experiment design. **B** The representative of HE staining, Masson’s trichrome staining, Picrosirius Red staining, and IHC staining for collagen I and fibronectin in kidney specimens from the indicated group of mice (*n* = 5 per group). **C** Quantitative analysis of Masson’s trichrome staining, Picrosirius Red staining, and IHC staining for collagen I and fibronectin in kidney specimens from the indicated group of mice. **D** The representative of IHC staining for NLRP3, CD3, and F4/80 in kidney specimens from the indicated group of mice. **E** Quantitative analysis of IHC staining for NLRP3, CD3, and F4/80 in kidney specimens from the indicated group of mice. **F** Representative images and quantitative analysis of DHE in kidney specimens from the indicated group of mice. **G** Representative images and quantitative analysis of TUNEL staining in kidney specimens from the indicated group of mice. *P < 0.05; **P < 0.01; ***P < 0.001; All data represent the mean ± SEM obtained from three independent experiments. Student’s *t* test.

To probe into the therapeutic potential of FIP1 in kidney injury in vivo, FIP1 was knocked down via adeno-associated virus-9 (AAV9) packed shRNA-FIP1, followed by construction of 14-day UUO (Fig. 7A). As demonstrated by Masson’s trichrome staining, Picrosirius Red staining and IHC staining, the UUO-induced kidney fibrosis was clearly alleviated by FIP1 KD (Fig. 7B, C). Additionally, the IHC staining for NLRP3 offered the in vivo evidence for FIP1-induced NLRP3 upregulation (Fig. 7D, E). Moreover, the infiltrating of T cells and macrophages was mitigated in FIP1 KD mice (Fig. 7D, E), reflecting the role of FIP1 in inflammation activation in vivo. Finally, the augmented levels of ROS and apoptosis in UUO kidneys were relieved by FIP1 KD (Fig. 7F, G).

### FIP1 KD alleviates IRI-induced inflammation activation, oxidative stress and apoptosis

**Fig. 8:**
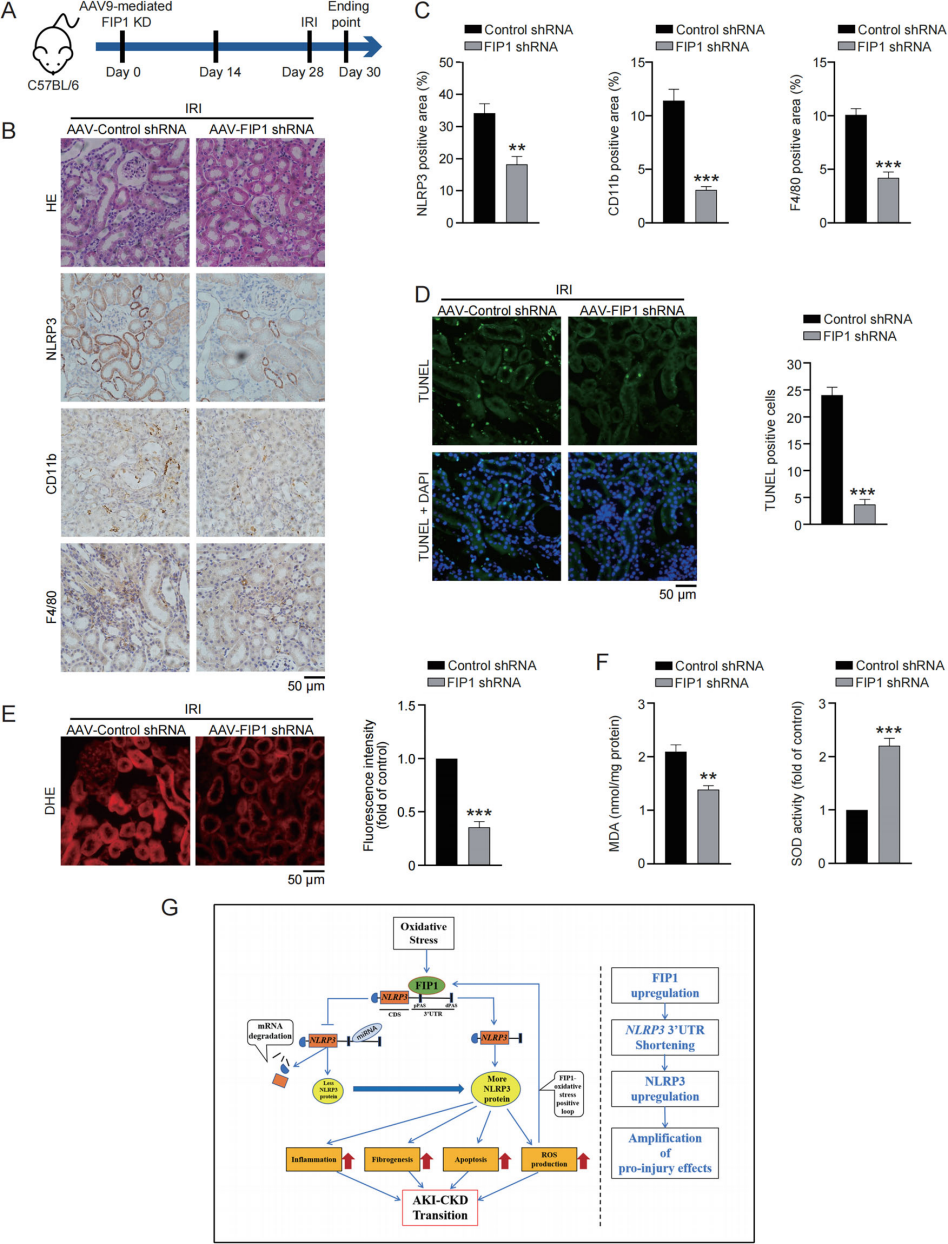
FIP1 KD alleviates IRI-induced inflammation activation, oxidative stress, and apoptosis. **A** Schematic diagram demonstrating the animal experiment design. **B** The representative of HE staining and IHC staining for NLRP3, CD11b, and F4/80 in kidney specimens from the indicated group of mice (*n* = 5 per group). **C** Quantitative analysis of IHC staining for NLRP3, CD11b, and F4/80 in kidney specimens from the indicated group of mice. **D** Representative images and quantitative analysis of TUNEL staining in kidney specimens from the indicated group of mice. **E** Representative images and quantitative analysis of DHE staining in kidney specimens from the indicated group of mice. **F** Quantitative analysis of MDA (left) and SOD (right) of mice from the indicated group. **G** Proposed model of FIP1-induced kidney injury progression and AKI-CKD transition. *P* < 0.05; **P < 0.01; ***P < 0.001; All data represent the mean ± SEM obtained from three independent experiments. Student’s *t* test.

We next examined the therapeutic potential of FIP1 in acute kidney injury in vivo, and the 2-day IRI was performed following FIP1 KD (Fig. 8A). IRI even induced a more significant upregulation of NLRP3 than UUO did, but FIP1 KD strongly reversed such NLRP3 upregulation (Fig. 8B, C). In particular, IHC staining for CD11b and F4/80 showed that FIP1 was instrumental in inflammation activation in IRI kidneys (Fig. 8B, C). Furthermore, less IRI-induced apoptosis was witnessed in FIP1 KD kidneys (Fig. 8D). Finally, DHE staining and measurement of MDA/SOD indicated the oxidative stress following IRI was reduced by FIP1 KD (Fig. 8E, F). Taken together, targeting FIP1 is a promising therapy for kidney injury.

## Discussion

Kidney injury is very much likely to transit into CKD and even kidney failure. Moderate inflammation is reno-protective role limiting the kidney damage. In contrast, amplified inflammation converts into an initiator of various pathological effects, especially apoptosis, oxidative stress, and fibrogenesis. Therefore, to inhibit the amplification of inflammation and subsequent pathological effects is an ideal strategy for kidney injury.

In most cases, NLRP3 upregulation is responsible for amplified inflammation and other pathological effects. To date, the upregulation of NLRP3 has been attributed to its enhancement of transcription or protein stability^34,35^. In this study, we reported for the first time that, APA-induced enhancement of *NLRP3* mRNA stability, is also a vital mechanism for NLRP3 upregulation. We found 3′UTR shortening stimulated NLRP3 upregulation via enhancing *NLRP3* mRNA stability. Such 3′UTR shortening-induced NLRP3 upregulation caused amplified inflammation, as well as enhanced apoptosis, ROS production and fibrogenesis, leading to kidney injury progression and AKI-CKD transition (Fig. 8G). These findings indicated 3′UTR shortening of *NLRP3* as a vital event of kidney injury progression, offering a new therapeutic strategy for kidney injury and other inflammation-associated diseases.

Oxidative stress is a well-recognized pathological factor in kidney injury, therefore a better understanding of how it exerts the po-injury effects in kidney will greatly benefit the development of treatment for kidney injury. In this study, the indispensable crosstalk between oxidative stress and APA was uncovered for the first time, especially in kidney injury progression. First, we found oxidative stress-induced upregulation of APA trans-factor FIP1 and further 3′UTR shortening of *NLRP3*, leading to amplification of multiple pathophysiological effects in kidney injury. It is worth noting that oxidative stress upregulated FIP1 expression, and in return FIP1 upregulation enhanced the oxidative stress. FIP1 thus formed a positive regulatory loop with oxidative stress in kidney injury progression. Second, inhibition of *NLRP3* 3′UTR shortening via silencing FIP1 strongly reversed the inflammation activation and apoptosis induced by oxidative stress. Based on the above evidence, this study built the first bridge between oxidative stress and APA. Besides, previous studies found oxidative stress-induced inflammasome activation via stimulating NLRP3 transcription^33^. Here we uncovered that via strengthening mRNA stability is another key mechanism for oxidative stress to promote NLRP3 upregulation and inflammasome activation.

Furthermore, we identified the APA trans-factor FIP1 as the upstream regulator governing 3′UTR shortening in kidney injury. We confirmed FIP1 predominantly bound to pPAS of *NLRP3* 3′UTR depending on its arginine-rich domain, inducing *NLRP3* 3′UTR shortening and NLRP3 overexpression. Moreover, FIP1 was strongly upregulated in UUO and IRI models. More importantly, FIP1 was overexpressed in clinical specimens of CKD and negatively associated with renal function of CKD patients. In vivo, FIP1 KD indeed alleviated the inflammation, oxidative stress, apoptosis, and fibrogenesis induced by UUO and/or IRI. FIP1 was found to be instrumental in self-renewal of stem cells, and no pathological role of FIP1 was identified. Therefore, this study uncovered its first pathological function. FIP1 may be a determinant of AKI-CKD transition and promising therapeutic target for kidney injury, highlighting the importance of the development of therapies targeting APA regulation, especially FIP1.

FIP1 is a well-known APA trans-factor, as well as the most important component of Cleavage and Polyadenylation Specificity Factor (CPSF) complex. Among the identified APA trans-factors, CFIm59, CSTF2, and FIP1 are the only ones proved to induce 3′UTR shortening. CSTF2 and CFIm59 unidirectionally function in favoring proximal PAS and inducing 3′UTR shortening. In comparison, of note, FIP1 was reported to favor distal PAS when the alternative PASs were close to one another^29^. However, inconsistent with the above-mentioned study, in the case of NLRP3, whose alternative PASs are close to one another, FIP1 still favored proximal PAS and induced 3′UTR shortening. Therefore, the actual function model of FIP1 in APA needs more intensive investigations.

Aberrant expression of NLRP3 is responsible for overactivated inflammation and subsequent onset of disease^35,36^. However, to date, NLRP3 upregulation is mostly found to be associated with enhancement of transcriptional activity or protein stability of NLRP3. In this study we first reported that enhanced mRNA stability derived from APA regulation, was also an important source of NLRP3 overexpression, suggesting to inhibit the 3′UTR shortening of *NLRP3* could be a promising therapeutic strategy, offering a novel insight to the development of NLRP3-targeted therapy.

In summary, this study identified APA regulation as a key role in kidney injury progression, as well as in inflammation overactivation. Additionally, this study offered FIP1 as a promising therapeutic target for kidney injury.

## Supplementary information

Supplementary Table
