# Supplementary figures and images for: Alternative polyadenylation trans-factor FIP1 exacerbates UUO/IRI-induced kidney injury and contributes to AKI-CKD transition via ROS-NLRP3 axis
Source: Cell Death Dis. 2021 May 19;12(6):512. doi: 10.1038/s41419-021-03751-3 (PMC8134587; doi:10.1038/s41419-021-03751-3)

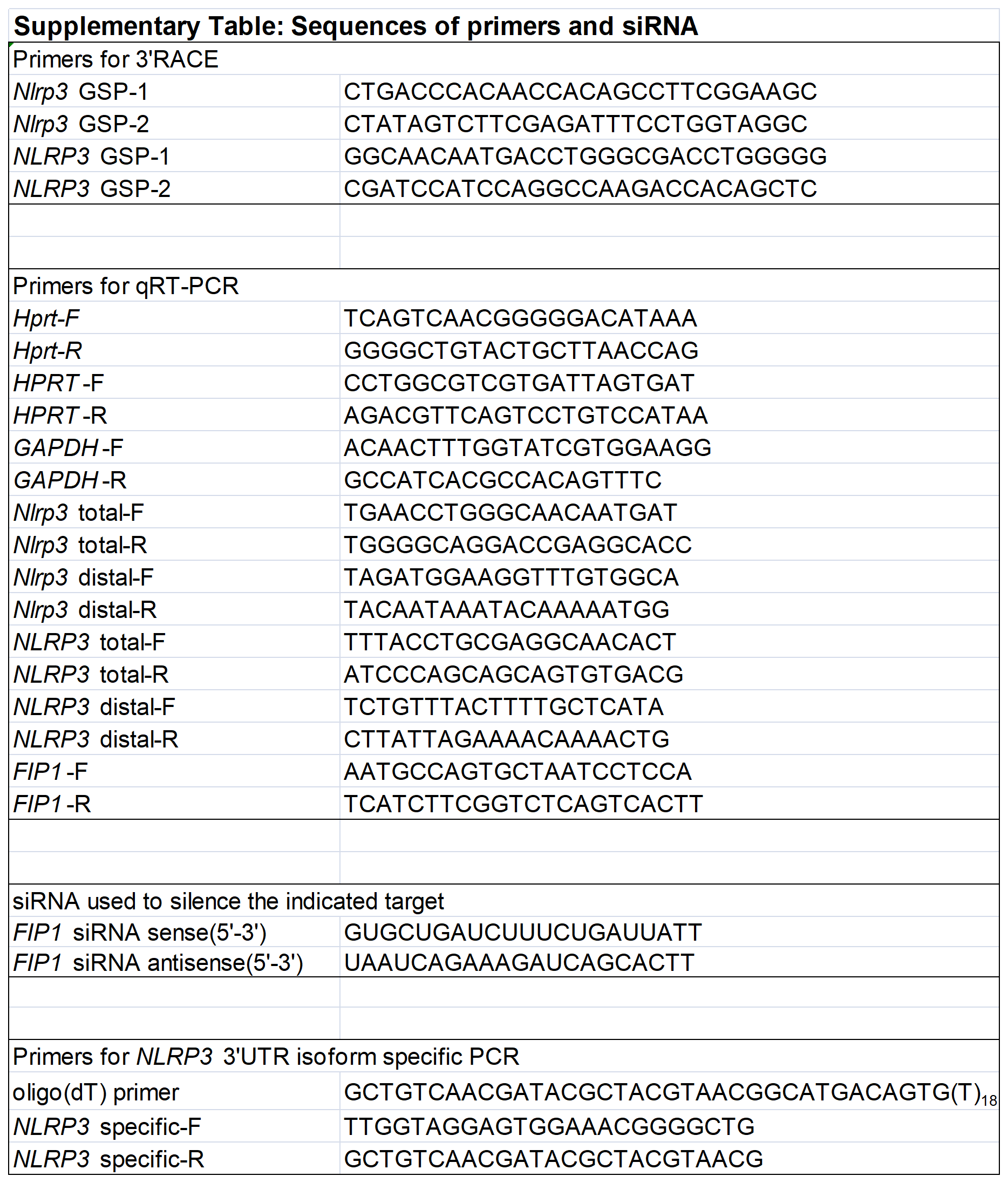

Supplement: Supplementary file 1 — Supplementary Table [file 41419_2021_3751_MOESM1_ESM.png]
